# Treatment of giant prostatic urethral stone with prostatolithotomy case report

**DOI:** 10.1016/j.ijscr.2023.109136

**Published:** 2023-12-20

**Authors:** Yunus Erol Bozkurt, Caner Buğra Akdeniz, Bilali Habeş Gümüş

**Affiliations:** a Manisa Merkez Efendi State Hospital, Department of Urology, Manisa, Turkey; b İzmir Foça State Hospital, Department of Urology, İzmir, Turkey; c Manisa Celal Bayar University Faculty of Medicine, Department of Urology, Manisa, Turkey

**Keywords:** Urethral stone, Giant stone, Prostate, Case report

## Abstract

**Introduction:**

The patient with prostatic urethral stones of the size mentioned in the case report is very rare, and there is no standard surgical procedure for patients with giant stones in the prostatic urethra.

**Presentation of case:**

A 62-year-old male patient presented to the emergency department with complaints of dysuria and hematuria. Computed tomography showed a prostatic urethral stone measuring 78x48x56 mm. Open prostatolithotomy was performed by extending the bladder incision towards the prostate capsule and the stone was removed.

**Discussion:**

Prostate stones can be classified into two types: true prostate stones, which form within the prostate's tissues, and urethral stones, which develop in the prostatic urethra. Urethral stones can be primary (forming in the urethra) or secondary (migrating from the upper urinary tract).

**Conclusion:**

Treatment options vary based on stone size and patient history, with endoscopy recommended as the primary approach. However, in cases with large stone burdens, open surgical methods may be preferred.

## Highlights

• Giant calculi in the prostatic cavity, though uncommon, pose unique diagnostic and management challenges.
• This case report presents a rare 78x48x56 mm prostatic urethral stone.
• Open prostatolithotomy was successfully performed, offering an alternative to endoscopic methods for large stones.
• This report provides valuable insights for urologists faced with similar rare and complex cases.

## 1 Introduction

Giant calculi in the prostatic cavity are uncommon, the diversity being those associated with chronic calculus prostatitis or small multiple calculi. Prostatic parenchymal calculi are usually incidental findings on computed tomography. They are asymptomatic and may be associated with benign prostatic hyperplasia and prostate cancer [1] Male urethral calculi is a rare clinical condition and is more common in Middle and Far Eastern societies, depending on socio-economic status and dietary habits. Most of these stones migrate from the bladder and upper urinary tract. In the literature, stone sizes ranged from 10 to 60 mm in a case series of 26 patients. [2] Various methods are used to treat urethral stones. In the case series of 14 patients, urethroplasty was performed in 5 cases, surgical removal of the stone in 3 cases, retrograde manipulation inside the bladder in 4 cases and electrohydraulic endourethral lithotripsy in 2 cases. [3] The global prevalence of urolithiasis and the existence of rare forms/types of urolithiasis in other parts of the urinary tract, in addition to the typical renal or ureteral occurrence, must not be forgotten. [11] We believe that this case report will guide the management of giant prostatic urethral stones.

## 2 Presentation of case

**Fig. 1:**
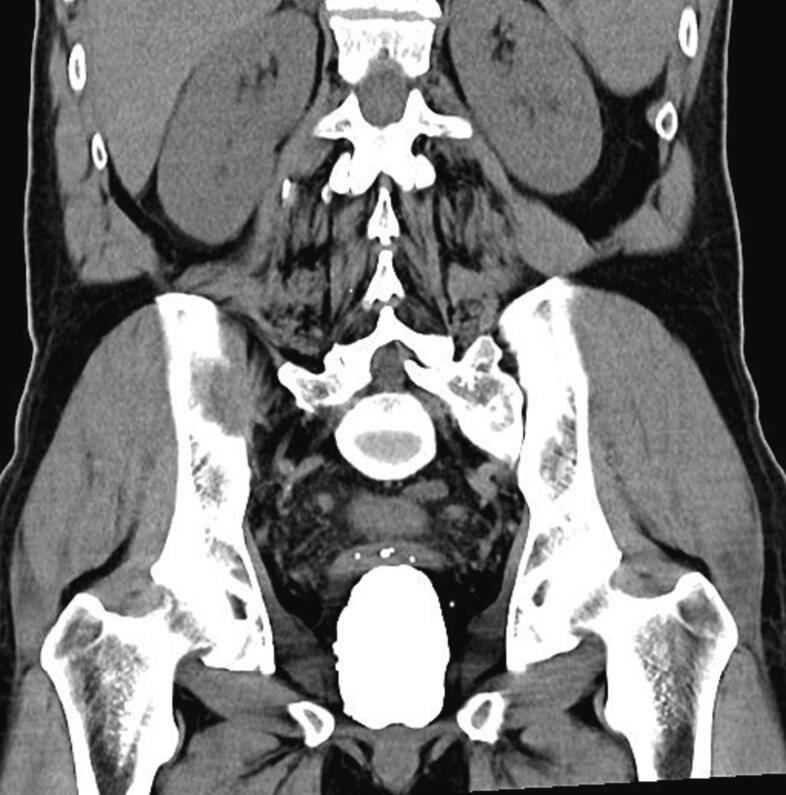
Preoperative tomographic imaging.

**Fig. 2:**
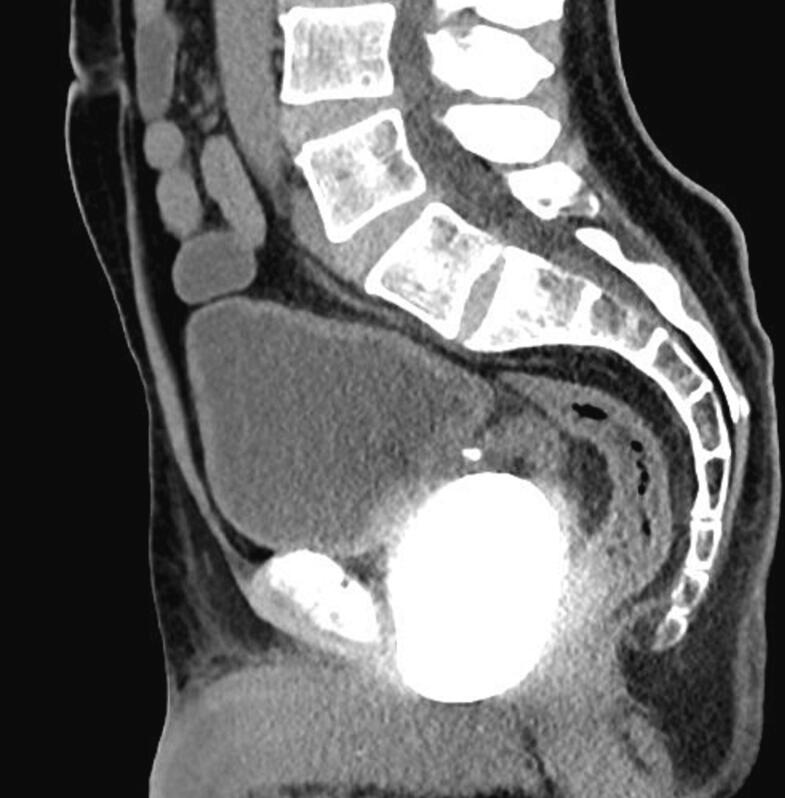
Preoperative tomographic imaging.

**Fig. 3:**
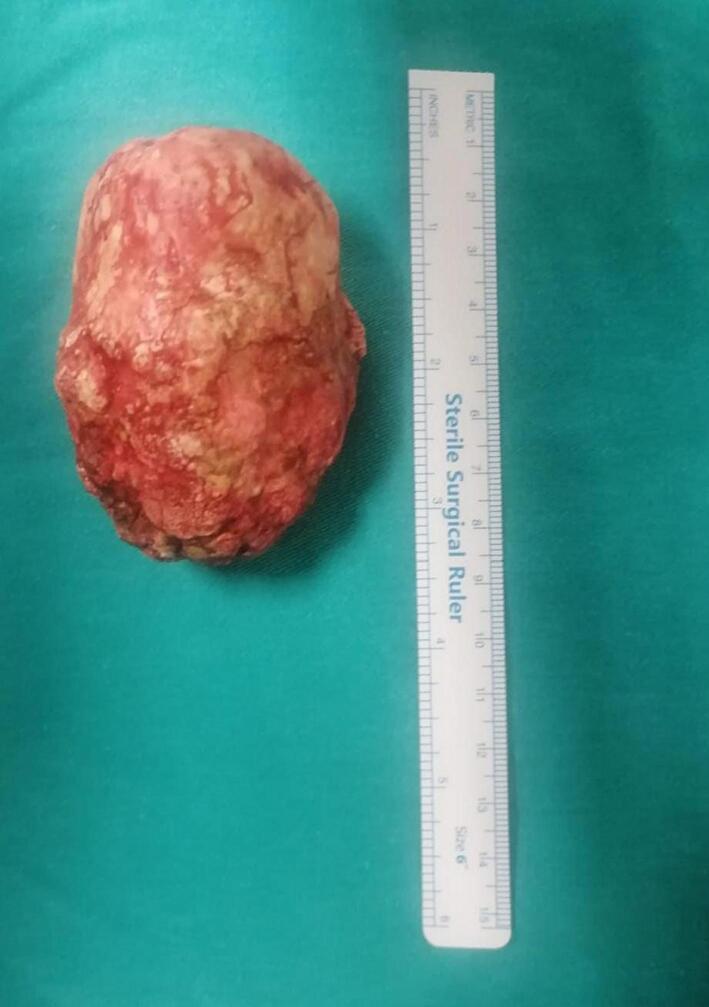
Post-operative view of prostatic urethral stone.

A 62-year-old male patient with no history of urological instrumentation or surgery was admitted to the emergency department with only complaints of dysuria and hematuria, but he had no history of fever, medication use for benign prostatic hyperplasia (BPH), acute urinary retention or urinary outflow obstruction. The patient had no family history of stones or prostate cancer. PSA measured 0.52 ng/ml. Since he had a history of stone expulsion about 20 years ago, plain abdominal radiography was performed first and then computed tomographic imaging was performed. As a result of imaging, a 78 × 48 × 56 mm prostatic urethral stone was detected (Fig. 1), but no dilatation in the upper urinary tract was observed (Fig. 2). On physical examination, the stone could be palpated in the perineal region, but no fistula tract was seen on the skin in this area. A prostatolithotomy was planned for the patient who was unable to undergo urethral catheterisation. An approximately 8 cm suprapubic sagittal incision (midline incision) was made and an open prostatolithotomy was initially performed. After coagulation and transection of some of the dorsal veins on the prostate capsule, the bladder incision was extended approximately 4 cm from the prostate capsule. Adhesions of the visualised stone to the prostate tissue were separated bimanually. The stone, grasped with an ovarian clamp, was removed from the prostatic urethra using perineal pressure and the bladder neck was sutured with 1–0 Vicryl. The extracted stone weighed 154 g (Fig. 3). A 22 French 3-way urethral catheter was placed in the bladder, bleeding was controlled and finally a drainage catheter was placed but no suprapubic catheter was used. There were no complications during or after the patient's 3-day hospital stay. Stone analysis could not be performed because the patient's health insurance did not cover it. Patient gave informed consent for publication. Our case was presented in line with SCARE guidelines [10].

## 3 Discussion

There are two types of prostate stones: true prostate stones and urethral stones. True prostate stones are those that develop in the acini or tissues of the prostate and should not be confused with urethral stones that develop in the prostatic urethra. Similarly, a stone in a diverticulum or an abscess associated with the urethra should not be considered a true prostate stone. Urethral stones are divided into primary and secondary: primary stones are those that form de novo in the urethra, and secondary stones are those that form in the upper urinary tract and migrate downwards. According to the history of our patient, the stone fits the definition of a secondary urethral stone [4]. The most common site of urethral stone is the posterior urethra. Although endoscopic treatment is recommended as the primary treatment in the literature, we preferred the open surgical method due to the high stone burden and prolonged operation time [5] Patients who remain asymptomatic for many years despite increased stone burden become rare cases when recognised in the late period, and whether to use the standard surgical procedure depends on the surgeon's experience [6]. A case report by Krystian Kaczmarek et al. presented a patient with a history of urethral surgery, a 7 cm prostatic urethral stone formed a perineal fistula, so the stone was treated by perineal incision [7]. Similarly, in the case report by Mustafa Kaplan et al., the stone was removed by perineal incision due to a urethrocutaneous fistula in a patient undergoing secondary catheterisation for spinal cord trauma [8].

Imaging and pathology of the male urethra is uncommon, but urologists make extensive use of endoscopic imaging. Computed tomography should not be neglected in patients with lower urinary tract symptoms, a history of urinary stones, and a history of previous urinary surgery [9].

## 4 Conclusion

This case report highlights the successful management of a rare case of giant prostatic urethral stone through open surgical intervention, demonstrating the importance of considering stone burden and individual patient characteristics when deciding on the surgical approach.

## Informed consent for publication

All authors have permission.

## Ethical approval

The patient's informed consent to participate was obtained for the case presentation. However, ethics committee approval was not obtained.

## Funding

None.

## Author contribution

YEB: Conceptualization, Data curation, Resources, Supervision, Validation, Visualization, Roles/Writing - original draft, and Writing - review & editing.

CBA: Investigation, Methodology.

BHG: Formal analysis, Project administration.

## Guarantor

Dr. Yunus Erol BOZKURT.

## Declaration of competing interest

None.

## Data Availability

All data generated or analysed during this study are included in this published article [and its supplementary information files].
